# Supplementary material for: Judgments of learning in bilinguals: Does studying in a L2 hinder learning monitoring?
Source: PLoS One. 2023 Dec 1;18(12):e0286516. doi: 10.1371/journal.pone.0286516 (PMC10691729; doi:10.1371/journal.pone.0286516)
Supplement: S1 File — (DOCX) [file pone.0286516.s001.docx]

Supporting Information – S1 File

Table S1. Participant information: Mean score (and standard deviations) for language measure.

|  | Experiment 1 | | Experiment 2 | | Experiment 3 | |
| --- | --- | --- | --- | --- | --- | --- |
| Self-reported measures | L1 | L2 | L1 | L2 | L1 | L2 |
| Daily exposure L1 (%) | 71.1 (14.7) | 23.2 (10.9) | 83.4 (17.4) | 40.3 (27.8) | 81.3 (18.0) | 33.2 (24.6) |
| Age of acquisition  (in years) |  | 7.9 (2.9) | 2.8 (1.7) | 5.8 (2.9) | 2.8 (1.3) | 7.0 (2.0) |
| Age of becoming fluent (in years) |  | 15.5 (5.0) | 5.1 (2.2) | 12.6 (3.2) | 5.3 (2.5) | 12.6 (2.7) |
| Speaking self-competence (0-10) |  | 7.6 (0.9) | 9.6 (0.9) | 7.8 (1.4) | 9.7 (0.7) | 7.7 (1.2) |
| Reading self-competence (0-10) |  | 8.2 (1.5) | 8.9 (0.9) | 8.9 (0.9) | 9.8 (0.4) | 8.4 (1.1) |
| Exposure to reading  (0-10) |  | 7.9 (2.0) | 8.6 (1.9) | 7.3 (2.2) | 9.1 (1.3) | 7.0 (2.4) |
| Learning by reading  (0-10) |  | 8.2 (1.5) | 9.2 (1.4) | 8.6 (1.4) | 9.5 (1.0) | 8.1 (2.0) |
| Language proficiency |  |  |  |  |  |  |
| MELICET (0-50 points) |  | 34.3 (7.2) |  | 36.2 (5.3) |  | 36.2 (4.6) |
| Verbal fluency L1 | 21.9 (4.1) | 15.3 (4.5) | 25.5 (4.2) | 18.0 (3.9) | 24.7 (6.6) | 16.3 (4.7) |

Verbal fluency task shows the mean number of words elicited in each language condition. By mistake, we did not record the data for L1 in the LEAP-Q in Experiment 1.

Table S2. Experiment 1: Estimated means (and standard deviations) for hits, false alarms, misses and correct rejections by language, font type and block order.

|  |  |  | Block order | | | |
| --- | --- | --- | --- | --- | --- | --- |
|  |  |  | L1-first | | L2-first | |
|  |  |  | Language | | Language | |
|  |  |  | L1 | L2 | L1 | L2 |
| Condition | Easy-to-read font | Hits | 0.90 (0.31) | 0.89 (0.32) | 0.86 (0.39) | 0.87 (0.38) |
|  |  | Misses | 0.09 (0.27) | 0.11 (0.29) | 0.13 (0.38) | 0.10 (0.35) |
|  |  | No response | 0.01 (0.33) | 0.01 (0.32) | 0.01 (0.24) | 0.03 (0.28) |
|  | Difficult-to-read font | Hits | 0.89 (0.32) | 0.88 (0.32) | 0.88 (0.38) | 0.85 (0.40) |
|  |  | Misses | 0.10 (0.29) | 0.12 (0.30) | 0.11 (0.36) | 0.13 (0.38) |
|  |  | No response | 0.01 (0.32) | 0.00^a^ (0.32) | 0.00^b^ (0.24) | 0.02 (0.26) |
|  | New items | Correct rejections | 0.91 (0.28) | 0.90 (0.30) | 0.79 (0.41) | 0.80 (0.40) |
|  |  | False alarms | 0.08 (0.26) | 0.09 (0.28) | 0.19 (0.39) | 0.17 (0.38) |
|  |  | No response | 0.11 (0.32) | 0.12 (0.32) | 0.07 (0.25) | 0.08 (0.27) |

Means of 0.00 is a matter of decimal places: a. 0.003125, b. 0.003378378.

Table S3. Experiment 2: Estimated means (and standard deviations) for hits, false alarms, misses and correct rejections by language, concreteness and block order.

|  |  |  | Block order | | | |
| --- | --- | --- | --- | --- | --- | --- |
|  |  |  | L1-first | | L2-first | |
|  |  |  | Language | | Language | |
|  |  |  | L1 | L2 | L1 | L2 |
| Condition | Concrete | Hits | 0.89 (0.31) | 0.90 (0.30) | 0.87 (0.33) | 0.91 (0.29) |
|  |  | Misses | 0.09 (0.28) | 0.09 (0.28) | 0.11 (0.31) | 0.07 (0.26) |
|  |  | No response | 0.02 (0.15) | 0.01 (0.09) | 0.01 (0.11) | 0.02 (0.12) |
|  | Abstract | Hits | 0.82 (0.39) | 0.85 (0.36) | 0.79 (0.41) | 0.86 (0.35) |
|  |  | Misses | 0.13 (0.34) | 0.14 (0.34) | 0.20 (0.39) | 0.12 (0.32) |
|  |  | No response | 0.05 (0.22) | 0.01 (0.12) | 0.02 (0.15) | 0.03 (0.16) |
|  | New items | Correct rejections | 0.85 (0.36) | 0.82 (0.38) | 0.84 (0.37) | 0.87 (0.33) |
|  |  | False alarms | 0.12 (0.33) | 0.16 (0.37) | 0.15 (0.35) | 0.10 (0.29) |
|  |  | No response | 0.03 (0.17) | 0.01 (0.11) | 0.02 (0.14) | 0.03 (0.17) |

Table S4. Experiment 3: Estimated means (and standard deviations) for hits, false alarms, misses and correct rejections by language, type of list and block order.

|  |  |  | Block order | | | |
| --- | --- | --- | --- | --- | --- | --- |
|  |  |  | L1-first | | L2-first | |
|  |  |  | Language | | Language | |
|  |  |  | L1 | L2 | L1 | L2 |
| Condition | Words grouped into semantic categories | Hits | 0.77 (0.42) | 0.87 (0.34) | 0.86 (0.35) | 0.91 (0.29) |
|  |  | Misses | 0.21 (0.41) | 0.12 (0.33) | 0.13 (0.34) | 0.09 (0.28) |
|  |  | No response | 0.02 (0.14) | 0.01 (0.10) | 0.01 (0.09) | 0.01 (0.10) |
|  | Unrelated words | Hits | 0.68 (0.47) | 0.73 (0.45) | 0.70 (0.46) | 0.86 (0.35) |
|  |  | Misses | 0.29 (0.45) | 0.27 (0.44) | 0.29 (0.45) | 0.14 (0.34) |
|  |  | No response | 0.03 (0.17) | 0.00^a^ (0.06) | 0.01 (0.10) | 0.01 (0.07) |
|  | New items | Correct rejections | 0.71 (0.45) | 0.81 (0.39) | 0.83 (0.38) | 0.86 (0.35) |
|  |  | False alarms | 0.26 (0.44) | 0.18 (0.38) | 0.17 (0.37) | 0.13 (0.33) |
|  |  | No response | 0.04 (0.18) | 0.01 (0.10) | 0.01 (0.10) | 0.01 (0.11) |

Means of 0.00 is a matter of decimal places: a. 0.004166667.

Table S5. Experiment 2: Concreteness ratings of each list. Mean (SD).

|  |  | Spanish – L1 | English – L2 |
| --- | --- | --- | --- |
| Mean (SD) concreteness rating | Concrete | 5.78 (0.5) | 4.56 (0.4) |
|  | Abstract | 3.76 (0.69) | 2.57 (0.67) |
|  | Total | 4.7 (1.18) | 3.64 (1.13) |
| Min. concreteness rating | Concrete | 4.8 | 3.64 |
|  | Abstract | 2.22 | 1.25 |
| Max. concreteness rating | Concrete | 6.66 | 5 |
|  | Abstract | 4.79 | 3.54 |

Note that concreteness ratings for English words were based on Brysbaert et al., (2014) using a 5-point scale, whereas values for Spanish words were based on LEXESP (Sebastián et al., 2000) using a 7-point scale. Thus, the descriptive statistics are in different scales. However, the criteria to consider a word abstract or concrete was equivalent for both data set as explained in the paper.

Table S6. Experiment 3: Frequency and number of letters of each study list. Mean (SD).

|  |  | Log10_FREQ | LEN_L |
| --- | --- | --- | --- |
| English–L2 | Semantic-category words | 1.5 (0.7) | 5.5 (2.0) |
|  | Unrelated words | 1.1 (0.5) | 5.5 (1.2) |
| Spanish–L1 | Semantic-category words | 1.3 (0.7) | 6.0 (1.7) |
|  | Unrelated words | 1.1 (0.5) | 5.8 (1.1) |

Log10_FRQ = mean estimated frequency; LEN_L = mean estimated number of letters. Unrelated words were used both for unrelated-word studied lists and for new words in the recognition test. All p values were above 0.05. (p>0.05) showing there was no significant difference.
